# Pain in the Ear

**Published:** 1898-03-05

**Authors:** Macleod Yearsley

**Affiliations:** Assistant Surgeon the Royal Ear Hospital, Surgeon-in-Charge of the Throat, Nose, and Ear Department of the Farringdon General Dispensary; formerly Clinical Assistant House Surgeon to the Royal Ear Hospital, &c.


### Medical Progress and Hospital Clinics.

[*The Editor will be glad to receive offers of co-operation and contributions from members of the profession. All letters should be addressed to* The Editor, at the Office, 28 & 29, Southampton Street, Strand, London, W.C.]

### PAIN IN THE EAR.

By Macleod Yearsley, F.R.C.S., Assistant Surgeon the Royal Ear Hospital, Surgeon-in-Charge of the Throat, Nose, and Ear Department of the Farringdon General Dispensary; formerly Clinical Assistant House Surgeon to the Royal Ear Hospital, &c.

### I.—Its Diagnosis.

Pain in the ear is a symptom for which the general practitioner is frequently consulted, and owns a multiplicity of causes. Upon an early and correct diagnosis often depends the success of treatment, since pain in the ear is as much a symptom of diseases of grave danger as it is of those of a trivial character; non-recognition of the cause of the pain may result in irreparable damage which might have been averted by prompt measures.

Pain may be referred by the patient to the ear, to the mastoid, or to parts of the head and neck around the ear; and it may take almost any form, from one of agonising intensity to one of simple dull headache. The accompaniments of constitutional disturbance and pyrexia are factors of great importance in diagnosis, and, when marked, render the pain of but secondary importance.

A classification of the causes of "ear-ache" and allied pains as a guide to differential diagnosis is best based upon the locality of the pain. Following this method all forms belong to one of three groups, and the following table will, I think, be found fairly comprehensive: I. Pain in the ear: (*a*) Without deafness or inflammation; (*b*) with deafness and inflammation. II. Pain in the mastoid: (*a*) With local swelling; (*b*) without local swelling. III. Pain outside the ear and mastoid process: (*a*) With acute suppurative otitis media; (*b*) with chronic suppurative otitis media. The causes of pain in or about the ear being thus conveniently tabulated, a detailed consideration of each group will enable their points of diagnosis to be better appreciated, and these may be discussed without further preface.

**I. Pain Referred to the Ear,** when occurring without deafness or inflammation, is usually due to some reflex cause, such as the irritation of a carious tooth, disease of the tongue or pharynx. In the great majority of cases the cause lies in the teeth. An interesting case came under my care two years ago; a boy, aged 13, complained of paroxysms of intolerable burning pain referred to the left meatus. Both ears were normal in appearance, and the hearing power was not impaired. The pain disappeared completely on the removal of two hypertrophied tonsils.

When pain is accompanied by deafness or inflammation, or both, it may be due to one of several causes. Occasionally, foreign bodies or collection of cerumen give rise to pain, either in the ear or referred to regions outside. Such cases are quickly recognised upon examination.

It is convenient to classify pain accompanied by deafness and inflammation into two groups, according as the external or middle ear is affected. In the former class of cases the impairment of hearing is generally moderate, and follows the pain. If the pain is not severe the meatus is red, swollen, tender, its walls bathed with a slight amount of discharge, and the introduction of a speculum is moderately painful; the condition is one of diffuse otitis externa. The symptoms of circumscribed otitis externa (aural furuncle) are much more marked. The pain is paroxysmal and severe, usually worse during the small hours of the night, waking the sufferer and preventing sleep. The introduction of the speculum causes much pain, and on inspection one or two rounded swellings are seen in the meatus.

When the pain is due to implication of the middle ear there is considerable deafness from the commencement. In making a diagnosis the amount of constitutional disturbance affords the best guide. In acute catarrhal otitis media and acute myringitis constitutional disturbance is slight, and pyrexia moderate. In the former disease the pain is severe, worse at night, aggravated by movement and by swallowing, and accompanied by the throbbing, hammering tinnitus caused by the pulsations of the internal carotid artery. The deafness is more marked than in simple myringitis, and is usually relieved by gentle Politzerisation. Acute myringitis, or inflammation of the tympanic membrane, occurring by itself is uncommon. It may be caused by the entrance of cold water into the ear in bathing, &c. When present the pain (as can be imagined from the structure of the membrane) is severe, deep-seated, extensive in character, and accompanied by throbbing and tinnitus. The pain is aggravated and the deafness increased by Politzerisation, Inspection shows the drumhead to be markedly congested, and pink in colour.

When the pain complained of is due to acute purulent otitis media the constitutional disturbance is marked, and the temperature runs high. In children, especially, the symptoms are highly suggestive of meningitis, a fact which marks the necessity of an examination of the ear whenever the latter disease is suspected. Often, indeed, the child will indirectly draw attention to the seat of its trouble by refusing to lie upon the affected ear. The membrana tympani will usually be found to bulge; there is often a yellowish tinge about the most prominent part; and, unless prompt measures be taken, spontaneous perforation will ensue, accompanied by an unnecessary degree of obstruction.

**II. Pain in the Mastoid.**—Pain in the mastoid, accompanied by local swelling, may be due to one of three conditions: Inflammation of a mastoid lymphatic gland, mastoid periostitis, or cortical mastoiditis. The first of these may be at once dismissed; the swelling is circumscribed and movable, and the pain is not great. The mastoid glands receive their lymphatics from the scalp behind the ear, and the cause of their swelling must, therefore, be sought outside the ear.

Mastoid periosititis is due, as a rule, to disease in the external meatus, and presents an immovable swelling, shelving down to the bone, and later becoming somewhat boggy.

Cortical mastoiditis, a condition of suppuration in the superficial cells of the mastoid, is a result of acute suppurative middle-ear disease. The swelling here is obviously of the bone, and is rapid in development. It is not of frequent occurrence.

Much more serious than the conditions just described is that of deep mastoiditis. A complication of chronic suppurative otitis media, occasionally also of the acute disease, it is one which calls for prompt relief. Pain (the most constant symptom) is of a deep throbbing, boring character. Cases of comparatively painless mastoid disease have, however, been recorded, but, being the exception, they may be here disregarded. Swelling is not infrequently absent, but when present is due to hyperæmia and œdema of the skin behind the ear, There is usually constitutional disturbance, sometimes marked, but rise of temperature is as often absent as present. One of the most important signs is a bulging of the superior posterior wall of the inner part of the meatus, and this, coupled with pain and a history of otorrhœa is, I think, sufficient to warrant prompt interference.

**III. Pain Outside the Ear and Mastoid Process.**—In this group are complications of ear disease which are most dangerous to the patient and difficult to the surgeon. They are those accompanied by severe constitutional disturbance and derangement of the cerebral functions. Unfortunately, in a paper like this, space does not allow of detail, and the best that can be done is to indicate in a few words the complications that may be expected and the method of detecting them.

Should there be pain diffused about the ear in the course of an acute suppurative otitis media, it may be relieved by performing paracentesis of the membrana tympani, with or without washing out the tympanum *viâ* the Eustachian tube. Should this proceeding not relieve the patient, and there is high temperature with retraction of the head, delirium, headache, vomiting, and constipation, the condition is the serious one of acute meningitis.

If, on the other hand, the surgeon has to deal with septic infection, the temperature will be of the oscillating "up and down" character, there will be a rigor, repeated within twenty-four hours, and the patient will exhibit the sallow, earthy look, the sweet breath, and may possibly become the subject of multiple abscesses.

In chronic suppuration of the middle ear a different set of complications present themselves. Of these four may be enumerated, all of them formidable. The patient may complain of occasional attacks of localised headache about the mastoid region. Such a complaint should always arouse suspicion as to the presence of chronic deep mastoiditis, in which the recurrence of the headaches is due to the hygroscopic swelling of collections of cholesteatomatous material. Unless surgically dealt with these cases may become infected by microorganisms and pass on to caries, cerebral abscess, or thrombosis of the sigmoid sinus.

Another complication, less easy of diagnosis, is that of subdural abscess. This may be suspected if there is continuous high temperature with localised swelling or tenderness on percussion. It cannot, however, be diagnosed with certainty, unless pus appears under the scalp or the trephine is used for purposes of exploration.

A third and very serious complication is that of phlebitis of the lateral sinus. The most important signs in this grave condition are an up and down temperature with an initial rigor, swelling and tenderness at the upper part of the internal jugular vein, and obstruction of the veins of the face on that side. The ophthalmoscope shows the retinal veins to be engorged, and usually there is present a tenderness over the exit of the mastoid emissary vein (the "post-mastoid tenderness" of Bennett).

Fourthly, the sufferer may be the subject of abscess, in which case the temperature will be found subnormal and the pulse slow. There is dull, localised headache, marked slowness of cerebration, and mental apathy deepening into insensibility. Optic neuritis is generally present and there is constipation. The abscess may be in either the cerebellum or the temporo-sphenoidal lobe, and whenever there is reason to suspect such a condition the surgeon should not hesitate to ascertain the real state of affairs by using the trephine; in the present day such a proceeding gives the patient a better chance (even if the diagnosis prove mistaken) than temporising.

