# Erratum

**Published:** 1898-03-12

**Authors:** 


Erratum.—In the former article, page 392, end of paragraph 9, for "obstruction" read "*de*struction."

